# *Moringa oleifera* Leaf Powder Dietary Inclusion Differentially Modulates the Antioxidant, Inflammatory, and Histopathological Responses of Normal and *Aeromonas hydrophila*-Infected Mono-Sex Nile Tilapia (*Oreochromis niloticus*)

**DOI:** 10.3389/fvets.2022.918933

**Published:** 2022-06-23

**Authors:** Seham El-Kassas, Nesreen Aljahdali, Safaa E. Abdo, Fatima S. Alaryani, Eman M. Moustafa, Radi Mohamed, Wesam Abosheashaa, Esraa Abdulraouf, Mohamed Atef Helal, Manal E. Shafi, Mohamed T. El-Saadony, Karima El-Naggar, Carlos Adam Conte-Junior

**Affiliations:** ^1^Animal, Poultry and Fish Breeding and Production, Department of Animal Wealth Development, Faculty of Veterinary Medicine, Kafrelsheikh University, Kafrelsheikh, Egypt; ^2^Department of Biological Science, College of Science, King Abdulaziz University, Jeddah, Saudi Arabia; ^3^Genetics and Genetic Engineering, Department of Animal Wealth Development, Faculty of Veterinary Medicine, Kafrelsheikh University, Kafrelsheikh, Egypt; ^4^Biology Department, Faculty of Sciences, University of Jeddah, Jeddah, Saudi Arabia; ^5^Department of Fish Diseases and Management, Faculty of Veterinary Medicine, Kafrelsheikh University, Kafrelsheikh, Egypt; ^6^Department of Aquaculture, Faculty of Aquatic and Fisheries Sciences, Kafrelsheikh University, Kafrelsheikh, Egypt; ^7^Department of Animal Wealth Development, Faculty of Veterinary Medicine, Kafrelsheikh University, Kafrelsheikh, Egypt; ^8^Department of Biological Science, Zoology, Faculty of Science, King Abdulaziz University, Jeddah, Saudi Arabia; ^9^Department of Agricultural Microbiology, Faculty of Agriculture, Zagazig University, Zagazig, Egypt; ^10^Department of Nutrition and Veterinary Clinical Nutrition, Faculty of Veterinary Medicine, Alexandria University, Alexandria, Egypt; ^11^Center for Food Analysis (NAL), Technological Development Support Laboratory (LADETEC), Federal University of Rio de Janeiro (UFRJ), Cidade Universitária, Rio de Janeiro, Brazil

**Keywords:** *Moringa oleifera*, dietary additives, phagocytosis, lysozyme level, inflammatory response, antioxidant activities, *A. hydrophila* infection

## Abstract

This study aimed to detect the impact of *Moringa oleifera* leaf powder dietary inclusion on the antioxidant and innate immune responses of mono-sex Nile tilapia fingerlings. A total of 180 fingerlings were allocated in a random method into three groups with triplicate each. One group (1^st^ group) received the control diet (basal diet (BD) free of moringa) and the other groups (2^nd^ and 3^rd^) fed BD containing *M. oleifera* leaf powder at 5 and 10% of the diet, respectively. After 6 weeks of feeding, fish were randomly redistributed into four replicates and rested for 24 h. Then, each fish in the first two replicates was injected with 0.2 mL of PBS, while the others were injected with 0.2 mL of *A. hydrophila* suspension (1.8 × 10^6^ CFU/mL). Healthy fish fed on *M. oleifera* leaf powder showed enhanced immune response manifested by significant increases in phagocytic and lysozyme activities with a marked H/L ratio (*P* < 0.05). In addition, significant alterations of the lymphocytic and heterophilic population in circulation with increasing infiltration in tissue such as the spleen were noticed. Also, *M. oleifera* significantly upregulated the antioxidants, *CAT* and *GPx*, proinflammatory cytokines, *IL1-*β*, IL-8*, and *IFN-*γ relative mRNA levels. On the other hand, following *A. hydrophila* challenging conditions*, M. oleifera* caused downregulations of *IL1-*β*, IL-8*, and *IFN-*γ transcription levels, and also lowered the *CAT* and *GPx* mRNA levels. In addition, a marked reduction of leukocytic infiltration plus a significant improvement of the degenerative changes in intestinal architecture has occurred. So, *M. oleifera* leaf powder can be included in the fish diet to enhance immune response under normal health conditions and lower the infection-associated inflammatory response.

## Introduction

Intensive aquaculture is commonly associated with increasing exposure to pathogens (1), causing serious economic losses and zoonotic threats (2, 3). Most of the fish pathogens, especially the bacterial ones, are normal inhabitants. Under stressful conditions such as sudden water temperature changes and increasing stocking density produce diseases and mass mortalities (4). Competing for these diseases depends on several traditional strategies, such as antibiotics or vaccines. However, antibiotic use is now dwindling due to antibiotic resistance (5–7) and the health risk to human beings (8). Besides, using vaccines is neither cost-effective nor practically applicable (2).

Consequently, recent research focuses on finding new alternative strategies that could reduce or replace antibiotics, prevent and control fish diseases, enhance environmental protection, and are safe for human and animal health (9–11). One of these alternatives is phytogenic immune-stimulant products that improve fish resistance by boosting their innate defense and antioxidant mechanisms (12). One of these products is the medicinal plants (13), which are cheap and have effective compounds that enhance fish immune response (14). These plants can be utilized in whole, in sections (roots, leaves, or seeds), or as extracts through inclusion in diet or water (13). *Moringa oleifera* (*M. oleifera*) is one of these plants cultivated in tropical and subtropical regions with multifunctional applications in agriculture, medicine, industry, and human and animal nutrition (15).

Different parts of *M. oleifera*, especially its leaves, are of high nutritional value, rich in poly-unsaturated fatty acids (16), essential amino acids, vitamins, and carotenoids, as well as have a low content of antinutrients like phytates, oxalates, saponins, and tannins (17, 18) besides they induce several biological activities such as antioxidant, hypolipidemic and immunomodulatory properties (19). *M. oleifera* leaf powder or its extract can be supplemented at 0.5, 1, or 1.5% without modulating fish's growth performance despite maintaining fish health under normal and in case of infection or toxicity (20–24). Moreover, its supplementation can be safely increased to 10% of the *Clarias gariepinus* (25) and *Oreochromis niloticus* (26) feed and 25% of a *Tilapia rendalli* feed (27) without harming fish health, and the most growth-enhancing level was 5% with a hypolipidemic effect (28).

The anti-stress properties of *M. oleifera* to alleviate stress either due to infection (22, 29) or toxicity (23, 24) in Nile tilapia have been proved to be mainly through modulating antioxidants response. Further, most studies were done on young fish (larvae) (22) and only investigated the antioxidant response. However, to our knowledge, the mechanism of how *M. oleifera* leaf powder modifies innate fish immunity at the transcriptomic level has not been investigated. So, the study aimed to explore how the dietary inclusion of *M. oleifera* leaf powder at 5 and 10% to mono-sex Nile tilapia fingerlings will modify its innate immune response under normal conditions and in case of *A. hydrophila* infection by examining phagocytic activity, lysozyme level, and transcriptional levels of some antioxidant and proinflammatory cytokines genes.

## Materials and Methods

### Fish Management and Experimental Design

The present trial was adopted on mono-sex Nile tilapia (*Oreochromis niloticus)* following the standard operating steps approved by the animal care committee (KFS-2019/9).

*O. niloticus* fingerlings, with an average body weight of 27.98 ± 0.75 g, *n* = 180, were acquired from a private farm. Fish was acclimatized for 14 days by feeding a commercial tilapia diet containing 30% Crude Protein with digestible energy of 4070 MJ/kg diet in glass aquaria (70 × 30 × 40 cm). An aerator and a multifunction internal filter (Shark SH-1000 Multi-Function Filter, China) were used for each glass aquarium. Following the acclimatization period, fish were allotted at random method into three different groups with triplicates each (20 fish/replicate). Groups' summary and descriptions are illustrated in Table 1. A control group (1^st^ group) was fed a basal diet (BD) free of *Moringa oleifera* leaf powder. While the 2^nd^ and 3^rd^ groups were fed BD containing 5% and 10% of *M. oleifera* leaf powder, respectively.

**Table 1:** Experimental design.

**Experimental design**												
Groups	G1 (Fed basal diet)				G2 (Fed basal diet contained 5% *Moringa oleifera* leaf powder)				G3 (Fed basal diet contained 10% *Moringa oleifera* leaf powder)			
Replicates	R1	R2		R3	R1	R2		R3	R1	R2		R3
Feeding period	Feeding trail included 2 weeks adaptation in in which fish were fed a commercial diet then, *Moringa oleifera* leaf powder contained diet was offered for 6 weeks.											
Challenging with *A.hydrophila*	R1	R2	R3	R4	R1	R2	R3	R4	R1	R2	R3	R4
	*no*	*no*	√	√	*no*	*no*	√	√	*no*	*no*	√	√
Sampling	Samples were collected after 24 and 48 h post *A.hydrophila* challenging											

*M. oleifera* leaves were thoroughly cleaned with water to get rid of dirt and dust, then air-dried at room temperature for 7 days or till complete drying. After that, a mechanical grinder ground moringa leaves into fine particles and stored them at 4°C until used in the diet formulation.

Diets (Table 2) were formulated and adjusted as indicated by (28). This feeding trial continued for 2 months. In the beginning, fish were supplied at a ratio equal to 4% of the body's weight, then decreased over the experiment until they reached 2.5 to 3% at the end of the feeding trial. The food was offered three times daily and the amount was changed every 2 weeks depending on the biomass in each aquarium. The aquarium water was partially (about 25–30%) changed with overnight stored water (dechlorinated water) 2 to 3 periods per week. Water quality parameters such as water temperature and dissolved oxygen (DO), total ammonia (NH_3_), total dissolved salts (TDS), pH, and electrical conductivity (EC) were determined based on methods previously used by El-Kassas et al. (28). Growth performance parameters and body indices were measured (Table 3) and published previously (28). However, they were included again in this study depending on a Copywrite agreement from Elsevier (License Number: 4983960289771).

**Table 2:** Ingredient's structure of the used trial feed.

**Ingredients (%)**	**Diet 1 (C)**	**Diet 2 (T)**	**Diet 3 (M)**
Corn oil	2.60	2.00	2.00
Wheat middling	8.25	7.30	7.30
Yellow corn	36.0	34.85	31.19
Soybean meal (44%)	28.0	26.0	27.0
Corn gluten meal (60%)	7.40	8.00	9.50
Carboxy methyl cellulose (CMC)	2.0	2.0	2.0
Moringa leaf powder		5.0	10.0
Fish meal(60%)	14.70	13.80	9.70
Mineral and vitamin premix (Premix)	0.30	0.30	0.30
CaHPO_4_	0.40	0.40	0.50
Methionine	0.05	0.05	0.10
Lysine -HCL	0.05	0.05	0.16
Choline chloride	0.05	0.05	0.05
Common salt	0.20	0.20	0.20
**Calculated analysis (%)**
Moisture	10.22	10.08	10.04
Ether extract	6.10	5.90	5.82
Crude fiber	3.80	4.10	4.19
Crude protein	30.0	30.05	30.04
Ash	6.20	6.10	5.90
Lysine	1.76	1.73	1.75
Methionine	0.68	0.67	0.68
Calcium	1.00	1.07	1.02
Phosphorus	0.88	0.85	0.77
NFE^*^	43.62	43.77	44.01
Digestible energy (DE Kcal/kg)^**^	2927.85	2934.90	2957.79

CaHPO_4_, contain Ca 21% and P 18%. Mineral and vitamin premix obtained from Allegaeu vet Co., Egypt, each kg includes: Fat soluble vitamins; vit K3 (3.333 mg), vit E (16.666 mg), vit D (666.666 IU), vit. A (3.333.333 IU); water soluble vitamins, vit B1 (3.333 mg), vit B2 (6.666 mg), vit B6 (5000 mg), vit B12 (6.6 mg), nicotinic acid (20.000 mg), pantothenic acid (13.333 mg), folic acid (1.666 mg), biotin (16.6 mg), elements, Fe (33.333 mg), Zn (16.666 mg), Mn (8.333 mg), Cu (5000 mg, I (100 mg), Se (100 mg), Co (66.6 mg), and CaCO_3_ as a carrier. Nitrogen-free extract (NFE^*^), ^**^Digestible energy, Carbohydrate (3.48 Kcal/g), Ether extract (8.5 kcal/g), Protein (4.49 Kcal/g), as (30). Tilapia diet supplemented with Moringa powder at 0, 5, and 10% levels were C, T, and M, diets, respectively.

**Table 3:** A summary of growth and water quality parameters (28).

	**C**	**T**	**M**	***P-*value**
Initial body weight (g)	27.68^a^ ± 0.74	27.84^a^ ± 0.77	28.42^a^ ± 0.80	0.7739
Final body weight (g)	50.53^b^ ± 2.74	61.00^a^ ± 2.52	48.67^b^ ± 2.59	0.0013
Final body weight gain (g)	20.13^b^ ± 2.57	33.16^a^ ± 2.47	21.38^b^ ± 2.62	0.0009
Feed intake/fish (g)	60.70^a^ ± 1.73	56.74^a^ ± 2.29	53.15^b^ ± 1.22	0.0497
Feed conversion ratio (FCR)	2.59^a^ ± 0.34	1.81^b^ ± 0.11	2.84^a^ ± 0.20	0.0021
Specific growth rate (SGR)	3.03^b^ ± 0.11	3.35^a^ ± 0.06	2.83^b^ ± 0.09	0.0006
Body length (cm)	14.69^b^ ± 0.30	15.53^a^ ± 0.21	14.62^b^ 0.25	0.0210
Body width (cm)	4.93^b^ ± 1.48	7.58^a^ ± 0.66	6.00^a^ ± 0.87	0.0411
Ammonia (mg/L)	0.052^a^ ± 0.001	0.048^b^ ± 0.002	0.0515^a^ ± 0.006	<0.0001
DO (mg/L)	4.76^b^ ± 0.10	5.24^a^ ± 0.20	5.34^a^ ± 0.14	0.042
pH	7.87^a^ ± 0.029	7.87^a^ ± 0.036	7.95^a^ ± 0.008	0.703
Temperature (°C)	27.13^a^ ± 0.118	27.20^a^ ± 0.107	27.06^a^ ± 0.230	0.824
TDS (mg/L)	7.87^a^ ± 0.029	7.87^a^ ± 0.036	7.95^a^ ± 0.008	0.703
EC (μS/L)	778.7^a^ ± 16.24	754.5^a^ ± 2.91	763.1^a^ ± 10.71	0.372

*C, T, and M, refer to diets used in this feeding trail. Moringa leaf powder was included at 0, 5, and 10% of C, T, and M diets, respectively. TDS, Total dissolved salts; EC, Electric conductivity; DO, dissolved oxygen. The results are expressed as means ± SEM (n = 3). Different superscript letters within the same row donate significant difference at p < 0.05 using one-way ANOVA*.

### Challenging With *Aeromonas hydrophila*

At the end of the feeding trial, fish in every group was redistributed in a random method and equally into four replicates (after confirming the non-statistical significance of replicating the effect, *P* > 0.05) and rested for 24 h to get rid of distribution's stress. Thus, fish in each treatment group with first and second replicates was injected with phosphate buffer saline (PBS). In contrast, the 3^rd^ and 4^th^ replicates infected with *Aeromonas hydrophila* strain were obtained. The severity of *A. hydrophila* was monitored routinely (31). *A. hydrophila* was cultured on tryptic soy broth (TSB) at 37 °C for 24 h with constant shaking (250 rpm) to obtain an inoculum of 1.8 × 10^6^ CFU/mL. The bacterial count was enumerated by standard dilution and plating assay. The median lethal dose (LD50) that kills 50% of injected fish was evaluated before the terminal injection challenge following (32). The challenge was conducted by intra-peritoneal injection of each fish with 0.2 mL fresh growth of *A. hydrophila* inoculum following (33). However, fishes in control negative ones were intra-peritoneally injected with 0.2 mL/fish of PBS. Mortalities were recorded daily.

### Sample Collection

Random fish samples (6 fish per aquarium) from each group after 24 and 48 h post-challenge were subjected to blood and tissue analysis. Fish were first mildly anesthetized with 100 mg/L of tricaine methanesulfonate (MS-222). Then, two blood samples (heparinized and non-heparinized) were collected through venipuncture of the caudal vein. The blood samples collected in heparinized syringes were used for measuring the differential leukocytic count and phagocytic activity. While blood collected without anticoagulant was used for serum separation (28) and was kept at −20°C for further analysis. Following blood collection, two spleen samples were collected from dissected fish. One sample was employed for qRT-PCR assay where it was kept in a sterile microcentrifuge tube, rapidly frozen in liquid nitrogen, and stored at −80°C for total RNA isolation. While the 2^nd^ spleen specimen was used for histopathological examination; thus, it was collected in a 10% neutral-buffered formalin fixative solution. In addition, samples from the mid-gut were collected, fixed overnight in a Bouin's solution, and employed for histopathological evaluation of intestinal morphometry. Lesion scoring analysis of lesions in the spleen and mid-gut was done according to (34, 35) with some modification in the scoring values. In addition, the number of goblet cells as an indicator of mucous production in the intestine was counted in five microscopic fields using a light microscope at a higher magnification. The goblet cells were recognized depending on whether they appeared white or clear when stained with H&E and are distributed in the epithelia of the intestine (36).

### Immunological Parameter Assessment

The differential leukocytic count was assessed following the method of Abo-Al-Ela et al. (37). Blood smears were briefly fixed with methanol and stained by rapid field stains (polychrome methylene blue and eosin). Then, it was rinsed with distilled water and subjected to air-dry. The blood film was examined using a computer-assisted light microscope under an oil immersion lens (×100). The number of heterophils and lymphocytes per every 100 leucocytes was counted and used to calculate the heterophils to lymphocytes (H/L) ratio.

Leucocyte phagocytic activity to *Candida albicans* was adopted *in vitro* (37). An aliquot of 100 μl of fresh blood sample was mixed with 100 μl of both *C. albicans* inoculum (1 × 10^6^ CFU/mL) and fetal bovine serum, then kept at 37°C for 30 min, afterward centrifuged at 1,500 rpm for 10 min. The blood smear was prepared with 5 μL of the resuspended cells. The phagocytic activity (PA) was estimated as the percentage of phagocytic cells which overwhelmed yeast cells. At the same time, the phagocytic index (PI) equaled the total number of phagocytized yeast cells classified by the count of phagocytic cells.

Measuring serum lysozyme activity depended on the agarose plate diffusion assay previously mentioned by Lie et al. (38). In brief, this method aimed to measure the diameter of clear lysed zones developed by different serum samples on 1% agarose gel containing a suspension of *Micrococcus lysodeikticus* cells (Sigma-Aldrich, 500 mg/L) and then compared to those formed by the standard (hen egg-white lysozyme solution, 20 mg/mL). In this regard, serum samples (25 μL) were inoculated in the agarose gel plate (1%) in a 50-mM phosphate buffer (pH 6.3). The plates were then closed tightly and kept at 37°C for 18 h. Serum lysozyme activity was analyzed using a logarithmic regression analysis according to this formula: Y = *A* + *Blog X*, where Y = diameter of the lysed zone and X = the lysozyme activity expressed in μg/mL.

### qRT-PCR Analysis of Some Antioxidant and Proinflammatory Cytokine Genes

According to the manufacturer's guidelines, total RNA was extracted from 50 mg of spleen tissues by Trizol (iNtRON Biotechnology, Inc.). The RNA integrity was confirmed by ethidium bromide-stained 2% agarose gel electrophoresis, while the concentration was estimated by Nanodrop (Uv–Vis spectrophotometer Q5000/Quawell, USA). Two micrograms of each RNA sample were utilized for cDNA synthesis by SensiFAST™ kit (Bioline, UK) according to the manufacturer's manual.

Real-time PCR was then performed to evaluate the relative mRNA transcriptomic profile for some antioxidant genes; catalase (*CAT*) and glutathione peroxidase (*GPX*) and some pro-inflammatory cytokines genes; interleukin 8 (*IL-8*), interleukin 1β (*IL-1*β), and interferon-gamma (*IFN-*γ), using specific primers (Table 4), in a Stratagene MX3000 system (Agilent Technologies), and SensiFast SYBR Low-Rox kit (Bioline, UK). PCR mixture preparations and the amplification conditions were done for each gene according to Dawood et al. (39). All genes tests were done in duplicates. CT values for each sample were estimated and combined in fold change (2^−ΔΔCT^) calculation according to Livak and Schmittgen method (40). In this regard, the relative transcription levels were normalized against the housekeeping gene (β*-actin*) and the corresponding values of the control negative group (fed BD free of moringa leaf powder and injected with PBS).

**Table 4:** Primer sequences.

**Gene**	**Primer**	**Reference**
*β-actin*	F: CAGCAAGCAGGAGTACGATGAG	Dawood et al. (39 )
	R: TGTGTGGTGTGTGGTTGTTTTG	
*CAT*	F: CCCAGCTCTTCATCCAGAAAC	
	R: GCCTCCGCATTGTACTTCTT	
*GPx*	F: CCAAGAGAACTGCAAGAACGA	
	R: CAGGACACGTCATTCCTACAC	
*IL-1β*	F: CAAGGATGACGACAAGCCAACC	
	R: AGCGGACAGACATGAGAGTGC	
*IL-8*	F: GCACTGCCGCTGCATTAAG	Abo-Al-Ela et al. (37)
	R: GCAGTGGGAGTTGGGAAGAA	
*IFN-γ*	F: AAGAATCGCAGCTCTGCACCAT	
	R: GTGTCGTATTGCTGTGGCTTCC	

### Statistical Analysis

The results were statistically analyzed by GLM procedure in IBM SPSS Statistics for Windows (SPSS version22, SPSS Inc., Il, USA) at *P* < 0.05. Before analysis, data were confirmed for normality and homogeneity of variance among variables by the Shapiro–Wilk and Levene test, respectively. Then, the results were subjected to three-way ANOVA to test the impacts of *M. oleifera* feed inclusion, challenge with *A. hydrophila*, and time of samples collected and their interactions. Using Tukey's HSD test, multiple comparisons were performed to determine the statistical significance. The results were expressed as means ± SEM. GraphPad Prism 9 statistical software (GraphPrism Software, La Jolla, California, USA) was employed to draw figures.

## Results

### Impact of *M. oleifera* Leaf Powder Dietary Inclusion on Immunological Parameters

**Figure 1:**
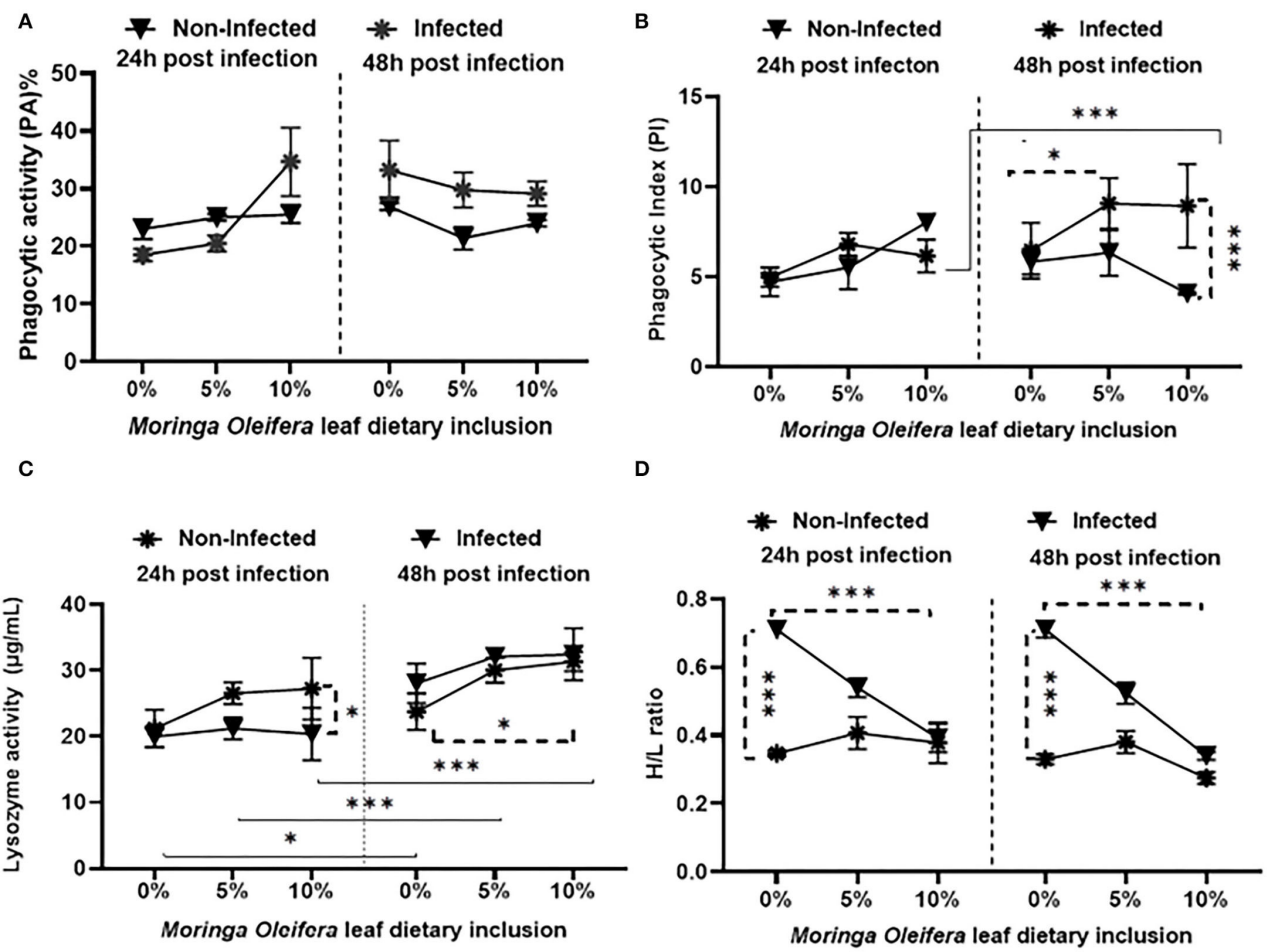
The effect of *M. oleifera* leaf powder dietary inclusion at 0, 5, and 10% of the diet on immunological parameters under normal health and following 24 and 48 h of *A. hydrophila* infection. **(A)** Represents the effect of *M. oleifera* inclusion on phagocytic activity (PA). **(B)** Shows the effect of moringa on the phagocytic index (PI). **(C)** Denotes the effect on lysozyme activity. **(D)** Shows the effect of moringa inclusion on the H/L ratio. Results were shown as means ± SEM. ^*^ and ^***^ denote *p* < 0.05 and 0.001, respectively.

Feeding on *M. oleifera* contained a diet that did not modify phagocytic activity (PA) (Figure 1A) either under normal or following *A. hydrophila* challenging conditions (*P* > 0.05). Only slight non-significant increases and decreases were measured in the case of 5% and 10% *M. oleifera* containing diet after 24 and 48 h post-challenge, respectively. On the other side, dietary feeding of *M. oleifera* to mono-sex Nile tilapia, challenging with *A. hydrophila*, time of sampling post challenge, and their interactions resulted in significant changes in the phagocytic index (PI) (Figure 1B) (*P* < 0.05). After 24 h post-challenge, no changes were noticed (*P* > 0.05). However, after 48 h, healthy fish fed on *M. oleifera-*contained diet (especially at 10%) showed a significant reduction of PI compared to those infected with *A. hydrophila* and fed on *M. oleifera-*contained diet (*P* < 0.05). In addition, among infected fish, a significant increase of PI was noticed in the case of those fed on a 5% *M. oleifera* compared to moringa-free diet (*p* < 0.05). Besides, increasing exposure to *A. hydrophila* resulted in a marked increase in PI (*p* < 0.05).

Feeding *M. oleifera* and exposure time to *A. hydrophila* resulted in a significant change in lysozyme activity (LZ) (Figure 1C) of mono-sex Nile tilapia (*p* < 0.05). Where, under normal health conditions, dietary inclusion of *M. oleifera*, especially at 10%, induced a significant increase in LZ activity (*p* < 0.05). LZ activity was significantly higher in infected fish than in the non-infected, especially 24 h after the challenge (*p* < 0.05). In addition, increasing the exposure time of mono-sex Nile tilapia to *A. hydrophila* significantly stimulated higher LZ activities in *M. oleifera* fed and non-fed groups (*p* < 0.05).

The effect of *M. oleifera* leaf powder dietary inclusion to mono-sex Nile tilapia on its differential leukocytic count and heterophil to lymphocyte (H/L) ratio is illustrated in Table 5 and Figure 1D, respectively. Healthy tilapias that were fed a moringa-containing diet exhibited significant increases in the lymphocytic count, with the highest count observed in the 10% moringa fed group (*p* < 0.05). In contrast, significant reductions in heterophils' count were recorded (*p* < 0.05). These changes in heterophils and lymphocyte count were associated with a significant reduction in the H/L ratio, which elevated with increasing moringa inclusion level in the diet (*p* < 0.05). In addition, Nile tilapias supplied with an *M. oleifera* leaf powder containing diet showed changes in the differential leukocytic count and H/L ratio following *A. hydrophila* infection. No changes were detected after 24 h of tilapia's challenge with A. hydrophila (*p* > 0.05). However, after 48 h of *A. hydrophila* exposure, a significant elevation was documented for the lymphocytic count in the case of the 10% moringa fed group (*p* < 0.05). At the same time, the heterophils count increased in the case of 5% moringa-fed fish (*p* < 0.05). These changes were also linked with lowering the H/L ratio.

**Table 5:** Differential leukocytic counts (%) of mono-sex Nile tilapia supplied with *Moringa oleifera* leaf including diet.

	**24 h post infection**					**24 h non-infected**				
	**Lymphocytes**	**Heterophils**	**Monocytes**	**Eosinophils**	**Basophils**	**Lymphocytes**	**Heterophils**	**Monocytes**	**Eosinophils**	**Basophils**
C	69.6 ± 0.81^**Aa**^	24.1 ± 0.79^**Ba**^	3.9 ± 0.06	1.6 ± 0.06	0.8 ± 0.09	55.0 ± 1.45^**Bb**^	39.0 ± 1.35^**Aa**^	3.7 ± 0.10	1.6 ± 0.05	0.8 ± 0.05
T	66.8 ± 2.33^**Aa**^	26.6 ± 2.28^**Ba**^	4.3 ± 0.05	1.6 ± 0.04	0.7 ± 0.07	60.9 ± 0.96^**Ba**^	32.8 ± 1.16^**Ab**^	3.9 ± 0.03	1.4 ± 0.06	0.9 ± 0.20
M	68.3 ± 2.60^**Aa**^	25.0 ± 2.59^**Aa**^	4.5 ± 0.04	1.7 ± 0.03	0.6 ± 0.04	67.5 ± 1.86^**Aa**^	26.3 ± 1.99^**Ac**^	3.9 ± 0.03	1.5 ± 0.03	0.8 ± 0.08
	**48 h post infection**					**48 h non-infected**				
	**Lymphocytes**	**Heterophils**	**Monocytes**	**Eosinophils**	**Basophils**	**Lymphocytes**	**Heterophils**	**Monocytes**	**Eosinophils**	**Basophils**
C	69.7 ± 1.18^**Ab**^	22.9 ± 1.16^**Bb**^	4.8 ± 0.09	1.7 ± 0.08	0.9 ± 0.09	54.8 ± 1.40^**Bc**^	38.9 ± 1.40^**Aa**^	3.9 ± 0.05	1.6 ± 0.00	0.8 ± 0.05
T	67.5 ± 1.52^**Ab**^	25.4 ± 1.56^**Ba**^	4.8 ± 0.08	1.7 ± 0.04	0.7 ± 0.05	61.5 ± 1.08^**Bb**^	32.1 ± 1.33^**Ab**^	3.9 ± 0.03	1.5 ± 0.03	1.0 ± 0.24
M	72.7 ± 1.01^**Aa**^	19.9 ± 1.00^**Bb**^	5.1 ± 0.06	1.7 ± 0.03	0.6 ± 0.04	70.0 ± 0.62^**Aa**^	23.7 ± 0.62^**Ac**^	3.9 ± 0.06	1.6 ± 0.07	0.8 ± 0.03

*C, T, and M refer to diets used in this feeding trail. Moringa leaf powder was included at 0, 5, and 10% of C, T, and M diets, respectively. Data were analyzed using thee-way ANOVA test and the results are expressed as means ± SEM (n = 6 fishes/replicate). Different lowercase letters in the same column indicate the differences between various Moringa oleifera inclusion levels at p < 0.05. Different uppercase letters within the same row indicate statistical significance between infected and non-infected treatments at P < 0.05*.

### *CAT* and *GPx* mRNA Transcripts Following *M. oleifera* Leaf Powder Dietary Inclusion and *A. hydrophila* Challenge

**Figure 2:**
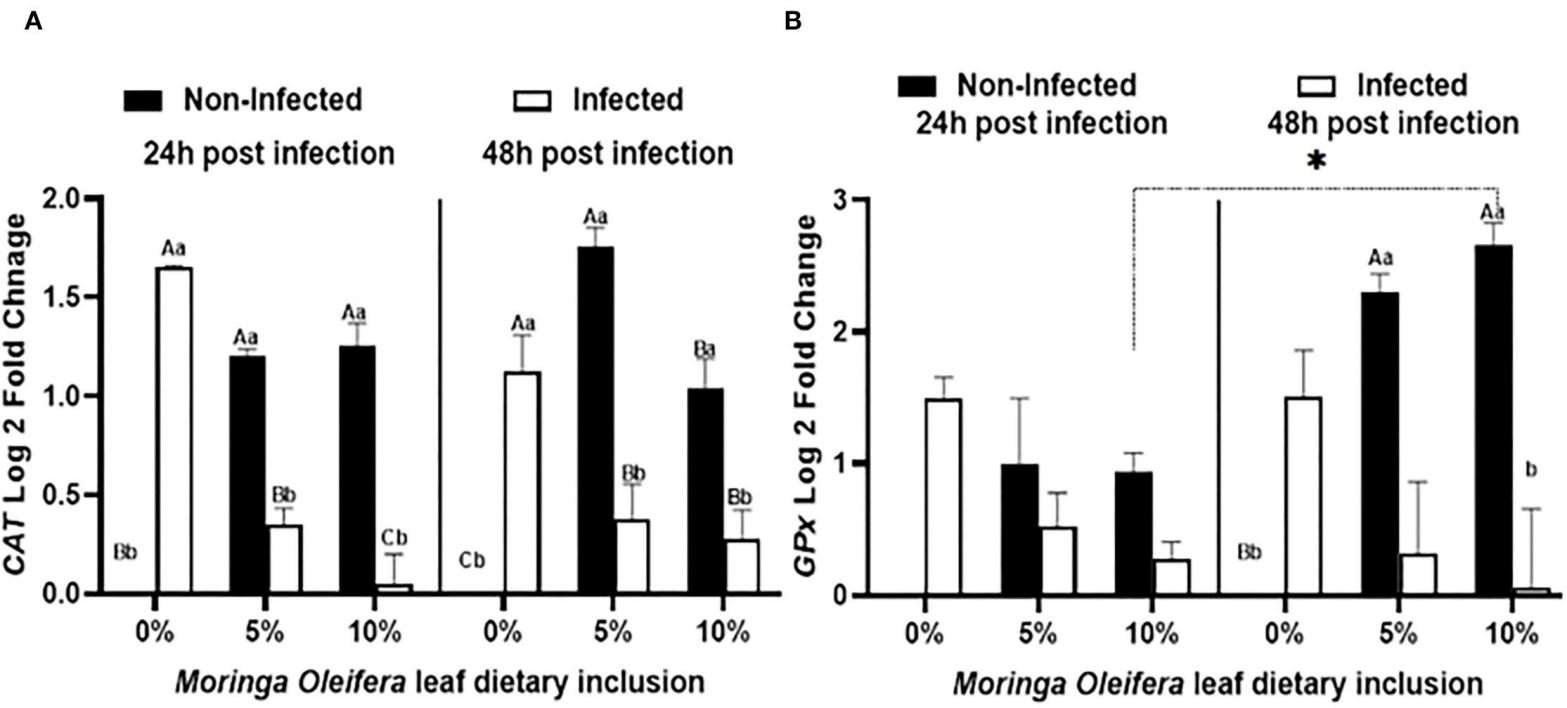
The relative mRNA expression level (6 fish/aquarium) of *CAT* and *GPx* in the spleen of mono-sex Nile tilapia fed dietary inclusion of moringa leaf powder at 0, 5, and 10% under non-infection and following 24 and 48 h post *A. hydrophila* infection. **(A)** Represents *CAT* log 2 fold changes and **(B)** shows *GPx* relative mRNA level (log 2 fold changes). Results are shown as means ± SEM. Different uppercase letters represent statistical significance at *P* < 0.05 between infection and non-infection conditions, while lowercase letters denote the statistical significance at *P* < 0.05 between different moringa inclusion levels. *P < 0.05.

Feeding moringa having a diet to mono-sex Nile tilapia modified the relative expression levels of some antioxidant genes such as *CAT* and *GPx* under normal health conditions and the infection with *A. hydrophila* after 24 and 48 h of exposure (Figure 2). Under normal health status, moringa leaf powder inclusion at 5 and 10% enhanced a significant upregulation of *CAT* relative mRNA level (*P* < 0.05). For *GPx* mRNA transcripts, feeding moringa leaf powder to healthy fish also stimulated higher levels and increased time (*p* < 0.05). On the other side, following infection with *A. hydrophila*, significant upregulations of both *CAT* and *GPx* were measured in the tilapias supplied with a moringa-free diet (0% moringa) (*p* < 0.05). The infection-induced upregulations were interestingly decreased in the case of moringa-fed groups, with the maximum reduction noticed in the 10% moringa and 48 h post-infection except for *CAT* which showed a slight increase after 48 h (*p* < 0.05). Therefore, *M. oleifera* feeding to mono-sex Nile tilapia stimulated a higher antioxidant response under normal health conditions, which decreased in case of infection.

### Proinflammatory Cytokines mRNA Transcripts' Response Following *M. oleifera* Dietary Inclusion and *A. hydrophila* Infection

**Figure 3:**
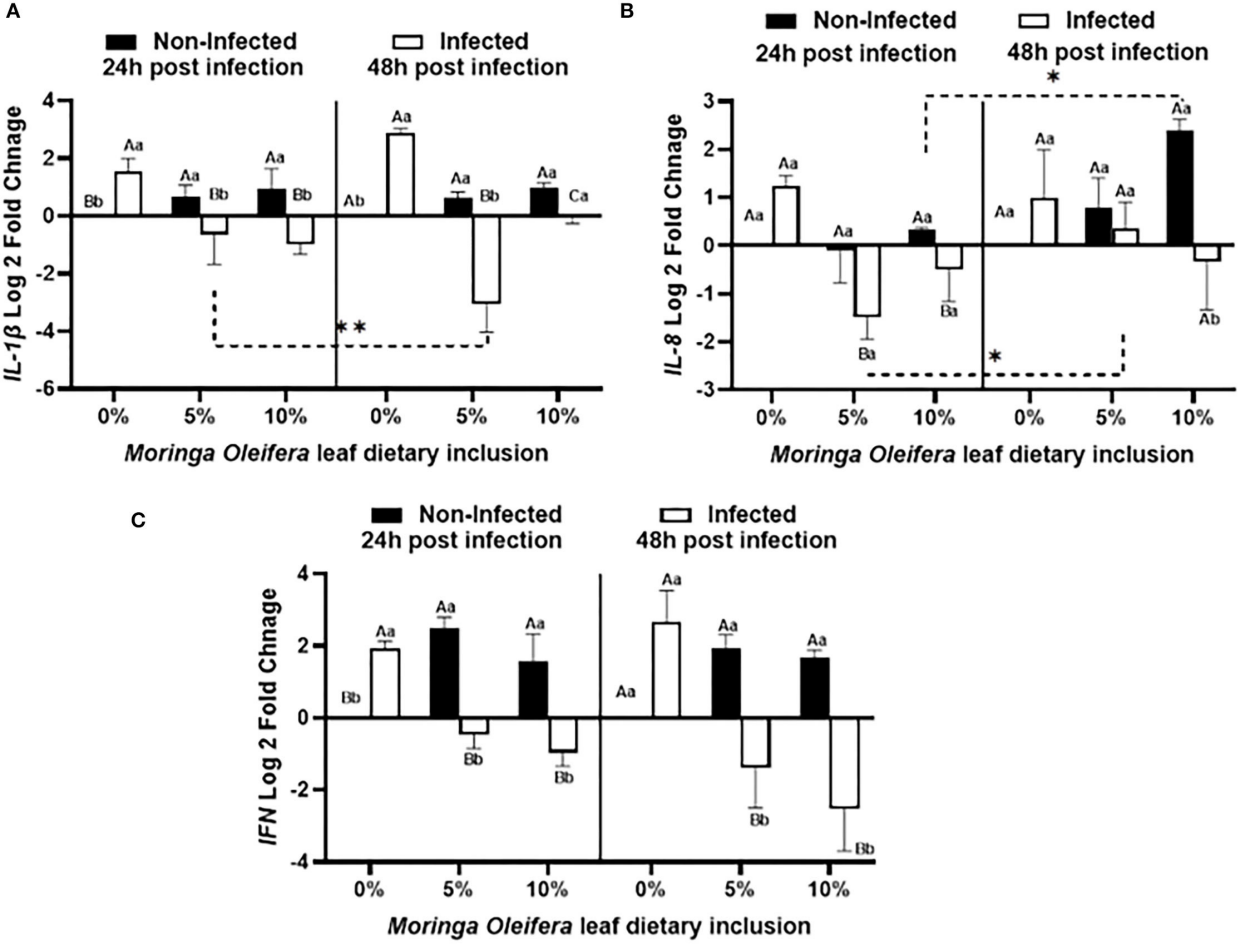
The relative mRNA expression level (6 fish /aquarium) of *IL1-*β, *IL-8*, and *IFN* in the spleen of mono-sex Nile tilapia fed moringa leaf powder dietary inclusion at 0, 5%, and 10% under non-infection and following 24 and 48 h post *A. hydrophila* infection. **(A)** Represents *IL1-*β log 2 fold changes. **(B)** and **(C)** Show *IL-8* and *IFN* relative mRNA levels (log 2 fold changes). Results are shown as means ± SEM. Different uppercase letters represent statistical significance at *P* < 0.05 between infection and non-infectious conditions, while lowercase letters denote the statistical significance at *P* < 0.05 between different moringa inclusion levels. *P < 0.001.

Dietary inclusion of *Moringa oleifera* induced significantly differential modulations of the relative mRNA expression levels of proinflammatory cytokines such as *IL-1*β*, IL-8*, and *IFN-*γ between normal health and infection conditions (Figure 3). For *IL-1*β (Figure 3A), normal healthy tilapias fed *M. oleifera* at 5% and 10 % inclusion levels exhibited significant upregulations of *IL-1*β in comparison with those supplied the BD (*P* < 0.05). However, in the case of *A. hydrophila* infection, fish fed moringa free diet showed a marked increased level of *IL-1*β. This effect was diversely changed in the case of fish fed on a moringa-containing diet and infected with *A. hydrophila* (*p* < 0.05). This downregulation increased with increasing the exposure time to *A. hydrophila*, especially in the case of the moringa 5% fed tilapias (*P* < 0.05). In addition, *IL-8* mRNA transcripts (Figure 3B) were significantly modified where, under normal conditions, moringa inclusion in the diet caused only slight increases of *IL-8* mRNA copies, which increased over time, especially in the case of the 10% moringa-fed group (*P* < 0.05). *A. hydrophila* infection caused a significant upregulation of *IL-8* expression levels in the absence of *M. oleifera* in the diet. This response was significantly downregulated in the case of moringa-fed groups (*P* < 0.05). In addition, this down-regulatory effect decreased over time, especially in the case of 5% moringa (*P* < 0.05). Likewise, *INF-*γ expression level (Figure 3C) was distinctly changed. Under normal health conditions, significant upregulations were reported in the case of moringa-fed groups compared to fish fed on BD free of moringa (P < 0.05). However, when fish fed on moringa-containing diet and infected with *A. hydrophila*, they displayed a significant downregulation to *IFN*-g relative mRNA level, which was distinctly increased following infection in the fish supplied with BD (*p* < 0.05).

### Histopathological Response of Mono-Sex Nile Tilapia Following *M. oleifera* Dietary Inclusion and *A. hydrophila* Infection

**Figure 4:**
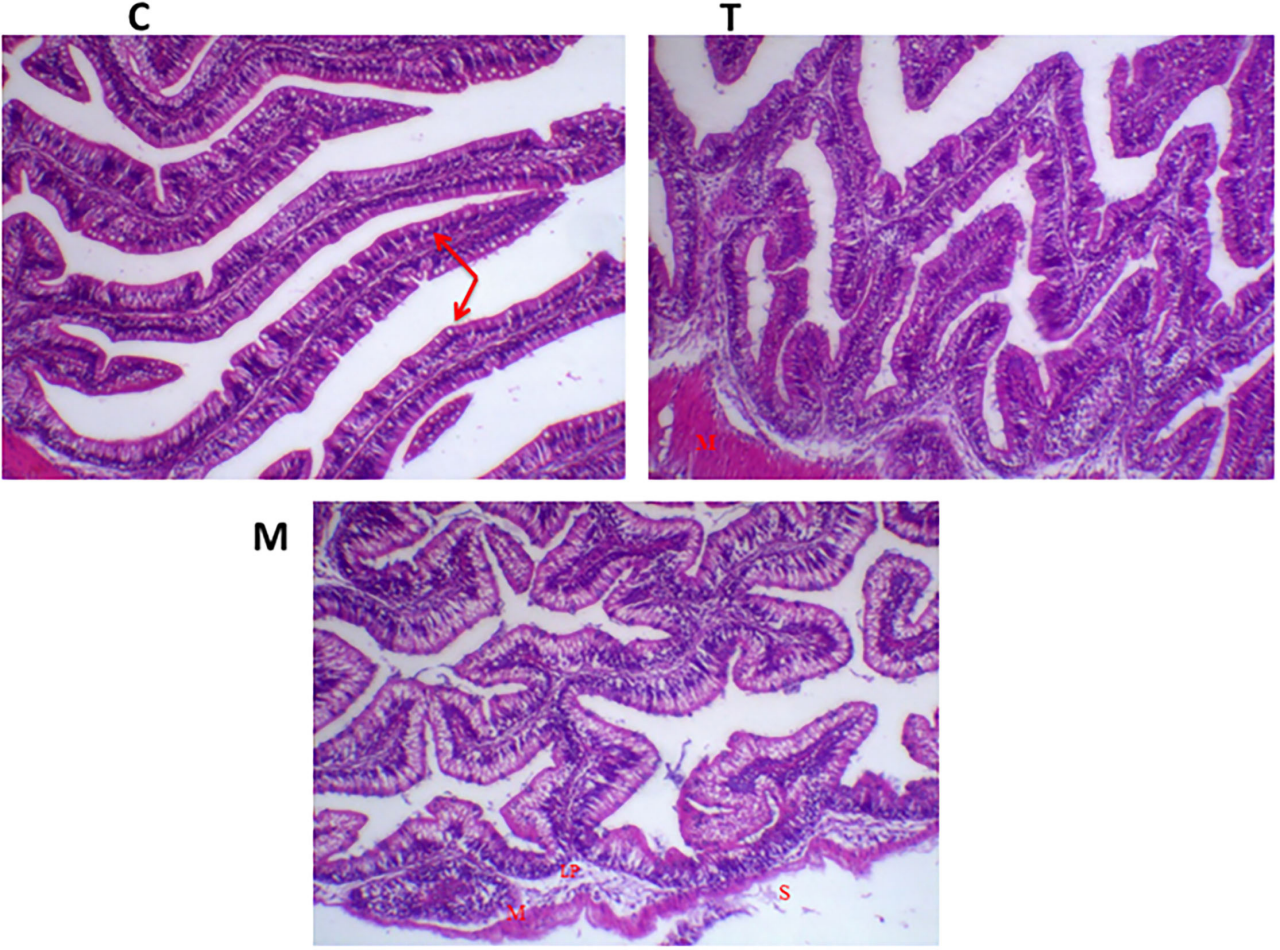
H&E stained photomicrograph of mono-sex Nile tilapia mid-gut following moringa leaf dietary inclusion for 8 weeks under normal health conditions. C, T, and M denote the 0, 5, and 10% moringa levels, respectively. Red arrow represents goblet cells, M represents tunica muscularis, LP means lamina probria sub-mucosa, while S means tunica Serosa.

**Figure 5:**
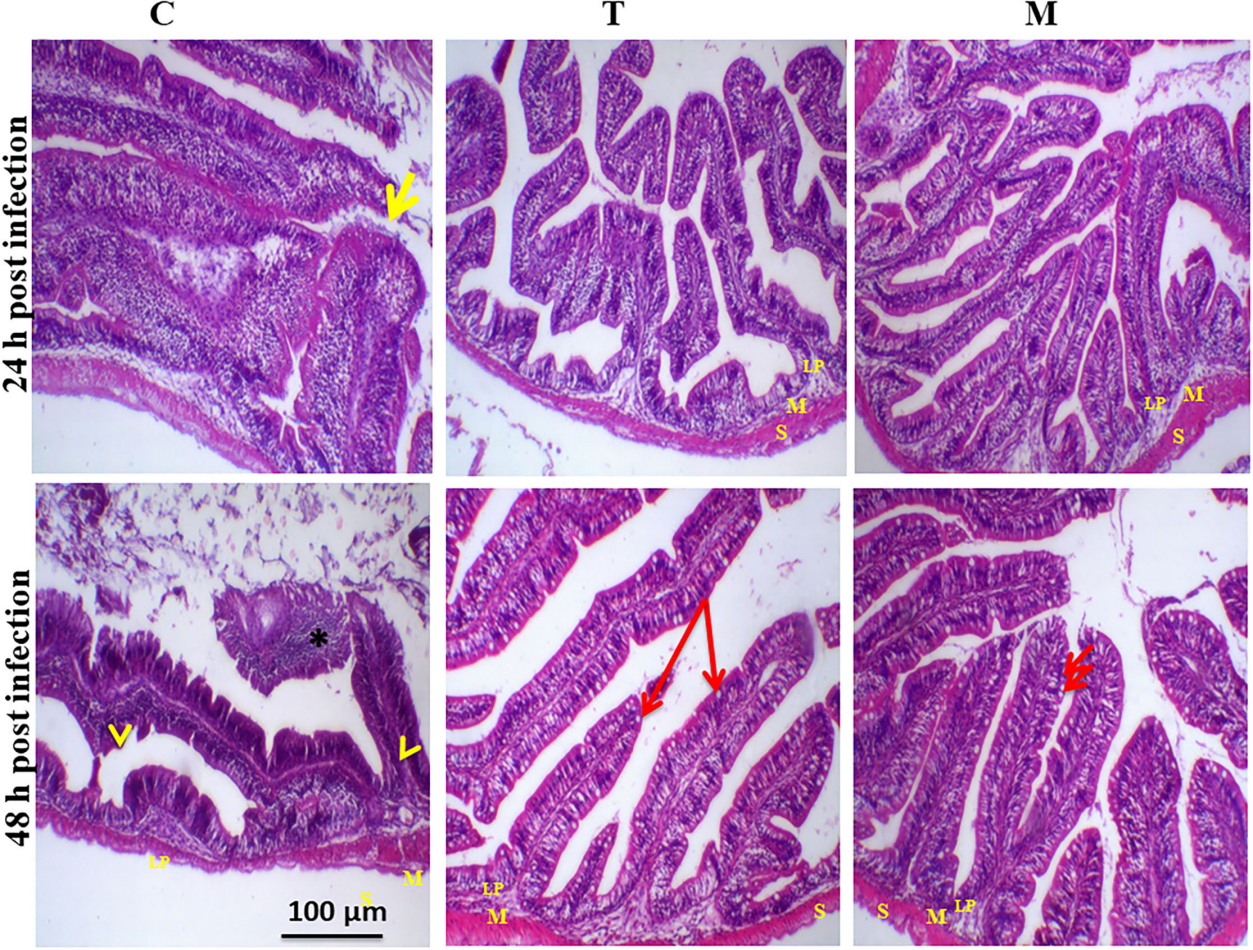
H&E stained photomicrograph of mono-sex Nile tilapia mid-gut following 24 and 48 h of *A. hydrophila* infection with and without moringa dietary inclusion. C, T, and M denote the 0, 5, and 10% moringa levels, respectively. The yellow arrow represents intestinal villi, yellow arrowhead indicates detachment of the apical part of enterocytes with leukocytic infiltration (star), while the red arrow represents goblet cells. M represents tunica muscularis, LP means lamina probria sub-mucosa, while S means tunica Serosa.

Histopathological changes in mid-gut from different groups were illustrated in Figures 4, 5 and Tables 6, 7. Under normal health conditions (Figure 4), fish fed the basal diet with 0% moringa showed normal and thin villi with intact four layers of mid-gut; tunica mucosa, lamina propria sub-mucosa, tunica muscularis, and tunica serosa. Dietary inclusion of *M. oleifera* increased villi length and branches of mid-gut with normal villi architecture (Table 6). In this regard, a significant dose-dependent increase in villi length and width was noticed, with the highest values reported 10% of moringa leaf powder. However, moringa leaf powder inclusion did not alter the inter villi space, with no pathological lesions noticed (0 scores). Infection with *A. hydrophila* caused marked degenerative changes in the mid-gut (Figure 5). After 24 h post-infection, clear blunting of intestinal villi (yellow arrow) was seen. In addition, increasing exposure to *A. hydrophila* (48 h post-infection) decreased villi length and caused detach of the apical part of enterocytes (yellow arrowhead) with leukocytic infiltration (star). These findings were scored over 2. On the other side, tilapias fed on *M. oleifera* at 5% and 10% showed intact and increased villi length with an increasing number of goblet cells (red arrow), especially with increasing the exposure time to infection (Table 8).

**Table 6:** Mid-gut morphometry of mono-sex Nile tilapia fed *M. oleifera* leaf powder containing diet.

	**24 h non-infected**			**24 h infected**		
	**Length (μM)**	**Width (μM)**	**Inter-villi space (μM)**	**Length (μM)**	**Width (μM)**	**Inter-villi space (μM)**
C	585.16 ± 32.05^Ac^*^^	90.19 ± 3.33^Ab^*^^	61.21 ± 3.69	466.90 ± 53.80^**Ab**^	60.69 ± 3.38^**Bc**^	53.63 ± 9.25
T	678.18 ± 21.88^**Bb**^	109.93 ± 4.95^Ab^*^^	73.39 ± 8.93	620.75 ± 22.95^**Ba**^	82.69 ± 9.78^**Bb**^	72.71 ± 4.32
M	830.67 ± 41.61^Aa^*^^	131.84 ± 15.17^**Aa**^	81.43 ± 16.58	616.66 ± 12.30^**Aa**^	111.59 ± 2.70^**Aa**^	69.36 ± 3.55
	**48 h non-infected**			**48 h infected**		
	**Length (μM)**	**Width (μM)**	**Inter-villi space (μM)**	**Length (μM)**	**Width (μM)**	**Inter-villi space (μM)**
C	568.95 ± 49.14^Ac^*^^	96.34 ± 4.26^**Ac**^	66.24 ± 4.48	474.89 ± 27.88^**Ab**^	93.49 ± 7.04^**Ab**^	62.13 ± 2.39
T	743.41 ± 18.08^Ab^*^^	118.58 ± 4.44^**Ab**^	80.51 ± 4.53	508.56 ± 23.11^**Ba**^	123.40 ± 3.51^**Aa**^	75.92 ± 2.39
M	851.39 ± 25.24^Aa^*^^	164.79 ± 25.32^**Aa**^	77.64 ± 4.85	558.87 ± 5.14^**Ba**^	169.53 ± 4.83^**Aa**^	69.88 ± 6.53

*C, control group fed diet with 0% M. oleifera; T, fishes fed M. Oleifera leaf dietary inclusion at 5% while M, fishes fed M. Oleifera leaf dietary inclusion at 10%. Data are presented as means ± SEM. Different letters represent statistical differences between different treatments at p- value < 0.05*.

* *P < 0.01*

**Table 7:** A summary of lesion scores in spleen and mid-gut of mono-sex Nile tilapia fed *M. oleifera* leaf powder containing diet.

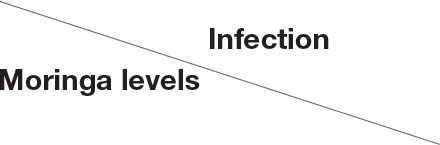	**Normal health condition**		**24 h post infection**		**48 h post infection**	
	**Spleen**	**Mid-gut**	**Spleen**	**Mid-gut**	**Spleen**	**Mid-gut**
C	00.00 ± 00^aB^	00.00 ± 00^aB^	2.83 ± 0.12^aA^	2.75 ± 0.14^aA^	2.87 ± 0.13^aB^	2.92 ± 0.08^aA^
T	00.00 ± 00^aB^	00.00 ± 00^aB^	1.90 ± 0.20^bA^	1.83 ± 0.22^bA^	1.60 ± 0.17^bA^	1.50 ± 0.14^bA^
M	0.00 ± 00^aB^	0.00 ± 00^aB^	1.30 ± 0.17^bA^	1.25 ± 0.14^bA^	1.30 ± 0.16^bA^	1.00 ± 0.63^bA^
***P*** **-values**	**Spleen**		**Mid-gut**	
Moringa effect	0.009		0.001			
Time effect	0.399		0.184			
Infection	0.026		0.004			
Moringa x time x infection	0.395		0.510			

*C, T, and M denote the 0, 5, and 10% moringa levels, respectively. Lesion scoring in spleen and mid-gut ranged from 0 to 3: 0 refers to non or slight pathological lesions; 1 refers to mild changes; 2, moderate changes; and 3 represents severe changes. Data are expressed as means ± SEM. Different letters represent statistical differences between different treatments at p- value < 0.05 using thee-way ANOVA test*.

**Table 8:** Goblet cells count (cell count/mm^2^) under normal health condition and following *A. hyrdophila* infection.

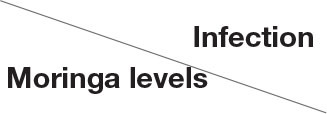	**Normal health condition**	**24 h post infection**	**48 h post infection**
C	142.00 ± 8.08^**cB**^	171.33 ± 6.96^**cA**^	168.67 ± 10.48^**cA**^
T	221.33 ± 11.62^**aB**^	242.67 ± 9.62^**aA**^	229.33 ± 7.06^**aA**^
M	196.67 ± 10.48^**bB**^	211.33 ± 14.44^**bA**^	221.33 ± 14.11^**aA**^
Moringa effect	*p* value <0.001		
Time effect	*p* value = 0.535		
Infection effect	*p* value = 0.045		
Moringa x time x infection	*p* value = 0.016		

*C, T, and M denote the 0, 5, and 10% moringa levels, respectively. Data are expressed as means ± SEM. Lower case letters represent the statistically significant effect of moringa, while upper case letters represent statistically significant effect of infection at P < 0.05 using thee-way ANOVA test*.

**Figure 6:**
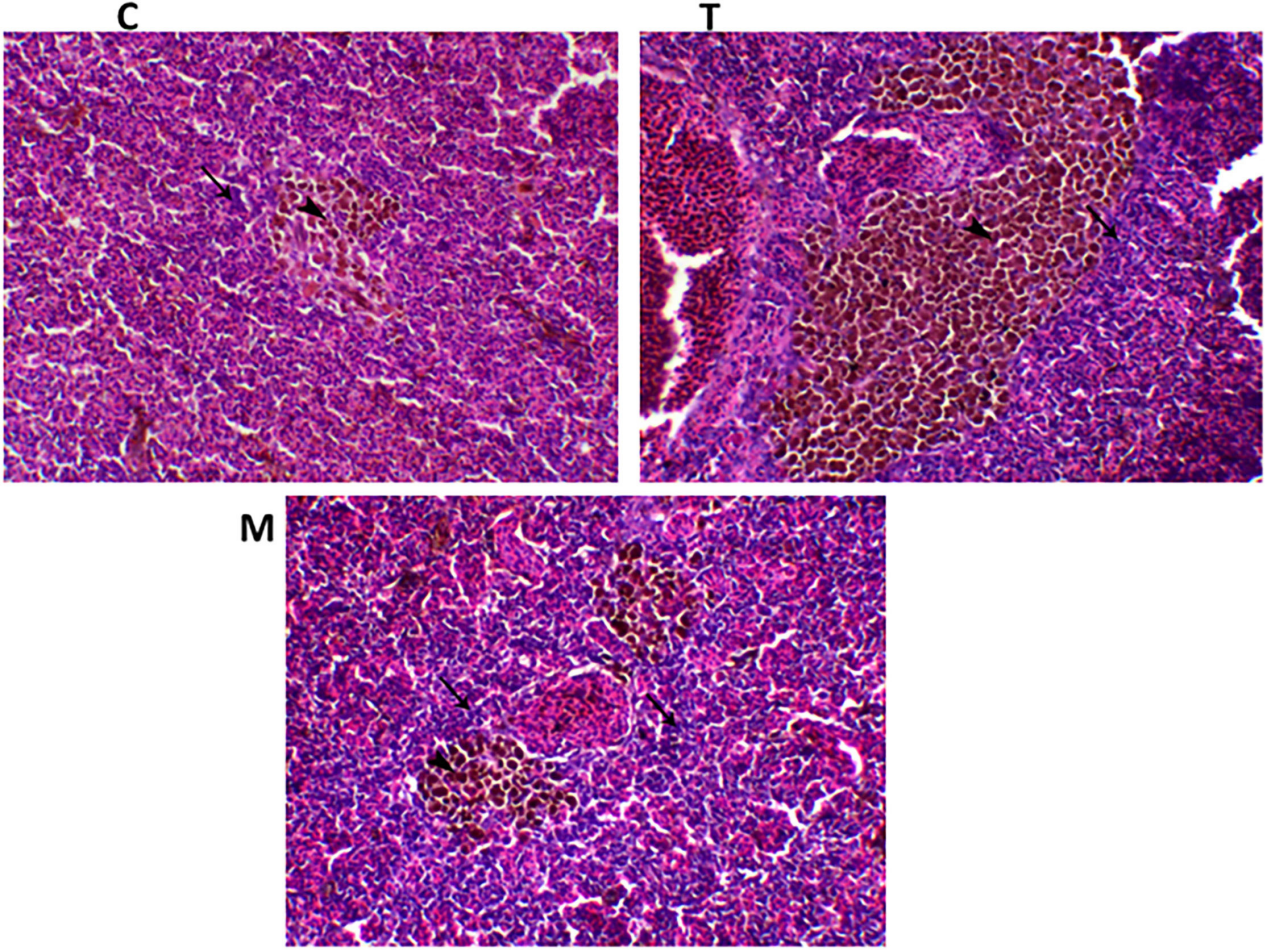
H&E stained spleen of tilapia following dietary inclusion of moringa leaf for 8 weeks under normal health conditions. C, T, and M denote the 0, 5, and 10% moringa levels, respectively. The black arrowhead represents melanomacrophage cells located within the white pulp, while the black arrow represents lymphocytic infiltration.

**Figure 7:**
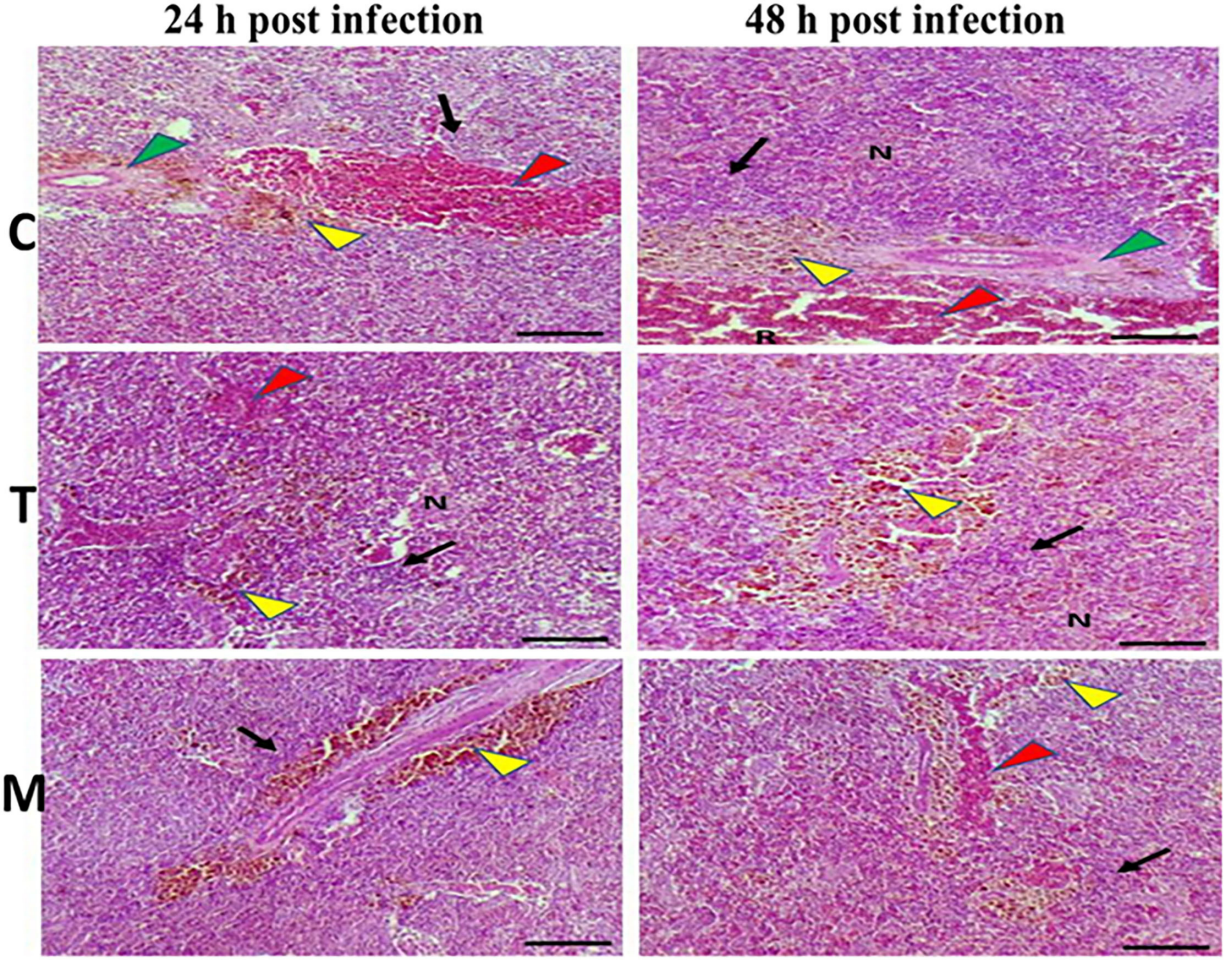
H&E stained histomicrograph of spleen of tilapia following 24 and 48 h of *A. hydrophila* infection with and without moringa dietary inclusion. C, T, and M denote the 0, 5, and 10% moringa levels, respectively. R, red pulp; W, white pulp; N, necrosis; red arrowhead (congestion), yellow arrowhead (Melanomacrophage center), green arrowhead (perivascular edema), black arrow (lymphocytic infiltration).

Spleen also displayed marked changes in its architecture which varied between normal and following infection conditions (Figures 6, 7). Under normal health conditions (Figure 6), tilapias fed on the basal diet free of moringa showed normal splenic tissue with normal red and white pulps with melanomacrophage cells located within white pulp (black arrowhead) and lymphocytic infiltration (black arrow). Interestingly, moringa inclusion at 5% and 10% of the diet caused marked hyperplasia of both melanomacrophage cells (black arrowhead) and increased lymphocytic infiltration within white pulp (black arrow). Since no pathological lesions were noticed, a 0 score was considered. On the other hand, after 24 and 48 h post *A. hydrophila* infection (Figure 7), the histological findings in the control groups showed signs of inflammation, including congestion of blood sinusoids and perivascular edema to degenerative changes in both red and white bulbs. The melanomacrophage center was present mainly near the blood sinusoids. These findings were confirmed with a significant increase in lesion scores (Table 7), where the highest score (around 3) was reported following *A. hydrophila* challenging and increased with increasing exposure time (*P* < 0.05).

Interestingly, the signs of inflammation improved with moringa inclusion, which caused decreased degeneration with very clear melanomacrophage centers and slight vascular congestion. The lymphocytic infiltration was revealed particularly after 48 h post-infection. Again, this effect was confirmed with significantly decreased lesion scores in moringa-fed groups and exposed to *A. hydrophila*.

## Discussion

The current study's foremost results revealed that the dietary inclusion of M. oleifera leaf powder in mono-sex Nile tilapias increased the phagocytic and lysozyme activities with a marked lowering of the H/L ratio, especially following *A. hydrophila* challenge. Similar findings were reported for *M. oleifera*, in many fish species, such as increasing serum lysozyme activity, immunoglobulin M (IgM) level, phagocytic, peroxidase, and the respiratory burst activities along with the complement component 3 (C3) values [reviewed by Abdel-Latif et al. (18)]. These immune-enhancing effects of *M. oleifera* might be due to its abundant contents of many biologically active phytochemicals such as polyphenols, volatile oils, vitamins, such as vitamin E, A, and ascorbic acid, phenolic acids, flavonoids, and sterols (β-sitosterol) with potential antioxidant, antifungal, and antibacterial properties [reviewed by Abdel-Latif et al. (18)]. On the other hand, the phagocytic activity and index were not influenced by moringa feeding under normal health conditions. This response concurs with (41–43), who recorded that the direct application of different plant extracts did not improve the leucocyte activities and even reduced them at larger doses.

Furthermore, following *A. hydrophila* challenge, the effect on PI increased with increasing time where there was no change after 24 h post-challenge, but after 48 h of the challenging, PI was significantly elevated. The latter effect might increase the dose of microorganisms that increased with increasing time (44).

Since lysozyme is an effective mucolytic innate immunity mediator of leukocytic origin that has antibacterial activity and stimulates phagocytosis, it is a good indicator of the non-specific immune functions of fish (45). In the present study, under normal health conditions, and following *A. hydrophila* challenge, moringa dietary inclusion induced a significant dose and time-dependent higher lysozyme activity. This increasing response is likely correlated with the antibacterial properties of *M. oleifera* due to its high content of several immune-stimulant bioactive components like carotenoids, moringinine, vitamins, minerals, sterols, alkaloids, flavonoids, caffeoylquinic acids, and phytoestrogens (41, 46). Our results agreed with (22, 46, 47) findings that reported increasing lysozyme activities with increasing moringa levels in the diet.

Besides, the aforementioned effects were accompanied by, under normal conditions, significant upregulations of the *CAT* and *GPx*, relative mRNA levels. These anti-stress properties of *M. oleifera* might be explained by its high content of phytogenic constituents, which protect against oxidative and stress-associated damages (48). In this regard, the high content of moringa leaf extract of ascorbic acid, phenolics, and flavonoids like sitosterol, quercetin, and kaempferol restore glutathione, glutathione-S transferase (GST), glutathione peroxidase (GPx), and glutathione reductase (GR) levels by preventing lipid peroxidation and scavenging free radicals (49–51). Besides reducing the lipid peroxidation, increasing the antioxidants such as SOD and CAT activities following dietary *M. oleifera* (48) is also probably linked with improving liver health (28).

Moreover, the antioxidant characteristic of moringa is likely mediated via the stimulation of nuclear factor (erythroid-derived 2)-like 2 (Nrf2) transcriptional factor, which has a critical role in stimulating multiple antioxidants and chemoprotective genes (52). The activation of Nrf2 is due to the high content of isothiocyanates in *M. oleifera* leaves (53). In turn, Nrf2 triggers the antioxidant response element (ARE), which upregulates the transcription of many genes such as GST, stimulating a higher GPx response (52–54). Our findings are consistent with (29) results, which reported that *M. oleifera* leaf extracts improved CAT, SOD, and GSH-Px activity in case of ammonia stress compared to the control.

While, in the case of *A. hydrophila* challenge, there was a marked upregulation of *CAT* and *GPx* transcripts which was significantly downregulated in the case of moringa fed groups. The noticed upregulation of *CAT* and *GPx* in the case of *A. hydrophila*-infected tilapias might be explained by *A. hydrophila* infection-induced stress response, which activates the Nrf2 response inducing more antioxidant synthesis mediated through ARE activation (52). Whereas the downregulated activities of *CAT* and *GPx* in the case of moringa-fed groups may be due to moringa bioactive components' direct potential antioxidant capabilities to inhibit the formation of hydroxyl radicals and stabilize free radicals or stabilize free radicals peroxyl-radicals because they are strong electron donors (hydrogen) (41). Also, because of their ability to neutralize free radicals or decompose peroxides into oxygen and water (55). Besides, lowering the *CAT* and *GPx* expression level is possibly associated with the anti-inflammatory characteristics of *M. oleifera* that reduces the reactive oxygen species (ROS) production due to reducing iNOS expression level, which was mediated by NF-κβ inactivation (52, 56).

In addition, the *M. oleifera* dietary inclusion stress relief response was associated with an alteration of leukocyte populations of the mono-sex Nile tilapias. Under normal health conditions, *M. oleifera* induced a dose-dependent increase and decrease in the lymphocytic and heterophils populations, respectively. This effect might explain the reduction of the H/L ratio. These results agreed with (57, 58) findings, which reported that aqueous moringa leaf extract stimulates a dose-dependent elevation in human lymphocyte viability and the count of CD4+ and CD8+ cells. On the other hand, following infection with *A. hydrophila*, there was no clear observed effect (only slight decreases) of moringa on leukocytic populations, which might correlate with moringa's properties to reduce the production of inflammatory mediators (41). This is probably confirmed by reducing the leukocytic infiltration in spleen tissue.

To the best of our knowledge, *Moringa oleifera* leaves have anti-inflammatory propriety, which is not well investigated at the transcriptomic level in Nile tilapia. Also, there are no available records on moringa leaves dietary inclusion on proinflammatory cytokines expression levels under normal health conditions. All available reports are on its impact on the inflammatory response in case of stress and infection-associated conditions. So, our study represents the first report on the effects of moringa leaves dietary inclusion on the transcriptomic profile of inflammatory mediator genes, such as *IL-1*β*, IL-8*, and *IFN-*γ under normal health and infection conditions. Under normal health conditions of mono-sex Nile tilapia, *M. oleifera* induced significant upregulations of the *IL-1*β*, IL-8*, and *IFN-*γ mRNA transcripts. This effect might be linked to increasing the number of lymphocytes and their infiltration in the spleen, which increased with increasing moringa levels in the diet. Moreover, this pro-inflammatory response might correlate with the high isothiocyanates content in moringa leaves (53). With its anti-inflammatory characteristics, it also promotes a pro-inflammatory response in some species, such as mice (59). The isothiocyanates associated with pro-inflammatory response are related to the activation of Th1 (59).

Besides, *A. hydrophila* challenge caused significant upregulations of *IL-1*β*, IL-8*, and *IFN-*γ mRNA levels which might be associated with the activation of the toll-like receptors (TLRs) pathway by LPS content of *A. hydrophila* cell wall (60). The latter activates NF-kB, which induces the expression of proinflammatory cytokines through a MyD88-independent pathway activation (60). The interesting reduction of this inflammatory response and downregulation of *IL-1*β*, IL-8*, and *IFN-*γ mRNA levels in the case of moringa-fed groups are perhaps linked with the inactivation of NF-κβ via Nrf2 activation by the high content of moringa leaves of isothiocyanates (53). Therefore, further future investigations of moringa dietary inclusion effects on Th2 and anti-inflammatory response are recommended. In addition, lowering the *A. hydrophila*-associated inflammatory response in the case of moringa-fed tilapias might clarify the mitigation of inflammatory signs in the intestine and the degenerative changes that occurred due to *A. hydrophila*.

Conclusively, *M. oleifera* leaf powder dietary inclusion enhanced mono-sex Nile tilapia's immune response under normal health conditions through increasing the phagocytic and lysozyme activities with upregulations of the CAT and GPx, and IL1-β, IL-8, and IFN-γ relative mRNA levels. Nevertheless, differential responses characterized by reducing the infection-associated inflammatory response as confirmed by downregulation of IL-1β, IL-8, and IFN-γ transcription levels and improving the degenerative changes in intestinal and spleen tissues were documented when fish were exposed to *A. hydrophila* and fed *M. oleifera* leaf powder diet. These differential responses may be explained by the different interactions of *M. oleifera* active components with fish physiological conditions, age, and the immune cascade to induce immune stimulation (41).

## Conclusion

The dietary inclusion of *M. oleifera* leaf powder to mono-sex Nile tilapia at 5% and 10% significantly improved the growth performance and modulated the immune response. The immune-modulatory response varied between normal health culturing conditions and in case of infection. Normally, *M. oleifera* stimulates the tilapia immune system manifested by increasing antioxidant activities, reducing stress H/L ratio indicator, and inducing a pro-inflammatory response distinguished by upregulation of *IL-1*β*, IL-8*, and *IFN-*γ transcripts. While, in the condition of infection, *M. oleifera* mainly acted to reduce the *A. hydrophila-*associated inflammatory and degenerative changes by downregulating the expression level of *IL-1*β*, IL-8*, and *IFN-*γ. Further investigations are suggested to deeply realize the exact mechanisms that mediate the *M. oleifera* effects between normal and diseased conditions.

## Data Availability Statement

The raw data supporting the conclusions of this article will be made available by the authors, without undue reservation.

## Ethics Statement

The animal study was reviewed and approved by the present trial was adopted on mono-sex Nile tilapia, *Oreochromis niloticus* following the standard operating steps approved by the Animal Care Committee, Faculty of Veterinary Medicine, Kafrelsheikh University, Egypt (KFS-2019/9). Written informed consent was obtained from the owners for the participation of their animals in this study.

## Author Contributions

SE-K, KE-N, and EM: conceptualization, data curation, resources, supervision, writing—original draft preparation, and writing—review and editing. RM, WA, CC-J, and EA: conceptualization, methodology, formal analysis, supervision, writing—original draft preparation, and writing—review and editing. MH and SA: data curation, investigation, and methodology. SE-K, KE-N, and EM: methodology and software. NA, MS, FA, ME-S, and EM: methodology and formal analysis. SE-K: methodology, software, and resources. KE-N: resources, data curation, and investigation. All authors contributed to the article and approved the submitted version.

## Conflict of Interest

The authors declare that the research was conducted in the absence of any commercial or financial relationships that could be construed as a potential conflict of interest.

## Publisher's Note

All claims expressed in this article are solely those of the authors and do not necessarily represent those of their affiliated organizations, or those of the publisher, the editors and the reviewers. Any product that may be evaluated in this article, or claim that may be made by its manufacturer, is not guaranteed or endorsed by the publisher.
