# Corrigendum: *Moringa oleifera* leaf powder dietary inclusion differentially modulates the antioxidant, inflammatory, and histopathological responses of normal and *Aeromonas hydrophila*-infected mono-sex Nile tilapia (*Oreochromis niloticus*)

**DOI:** 10.3389/fvets.2022.1127710

**Published:** 2023-01-25

**Authors:** Seham El-Kassas, Nesreen Aljahdali, Safaa E. Abdo, Fatima S. Alaryani, Eman M. Moustafa, Radi Mohamed, Wesam Abosheashaa, Esraa Abdulraouf, Mohamed Atef Helal, Manal E. Shafi, Mohamed T. El-Saadony, Karima El-Naggar, Carlos Adam Conte-Junior

**Affiliations:** ^1^Animal, Poultry and Fish Breeding and Production, Department of Animal Wealth Development, Faculty of Veterinary Medicine, Kafrelsheikh University, Kafrelsheikh, Egypt; ^2^Department of Biological Science, College of Science, King Abdulaziz University, Jeddah, Saudi Arabia; ^3^Genetics and Genetic Engineering, Department of Animal Wealth Development, Faculty of Veterinary Medicine, Kafrelsheikh University, Kafrelsheikh, Egypt; ^4^Biology Department, Faculty of Sciences, University of Jeddah, Jeddah, Saudi Arabia; ^5^Department of Fish Diseases and Management, Faculty of Veterinary Medicine, Kafrelsheikh University, Kafrelsheikh, Egypt; ^6^Department of Aquaculture, Faculty of Aquatic and Fisheries Sciences, Kafrelsheikh University, Kafrelsheikh, Egypt; ^7^Department of Animal Wealth Development, Faculty of Veterinary Medicine, Kafrelsheikh University, Kafrelsheikh, Egypt; ^8^Department of Biological Science, Zoology, Faculty of Science, King Abdulaziz University, Jeddah, Saudi Arabia; ^9^Department of Agricultural Microbiology, Faculty of Agriculture, Zagazig University, Zagazig, Egypt; ^10^Department of Nutrition and Veterinary Clinical Nutrition, Faculty of Veterinary Medicine, Alexandria University, Alexandria, Egypt; ^11^Center for Food Analysis (NAL), Technological Development Support Laboratory (LADETEC), Federal University of Rio de Janeiro (UFRJ), Cidade Universitária, Rio de Janeiro, Brazil

**Keywords:** *Moringa oleifera*, dietary additives, phagocytosis, lysozyme level, inflammatory response, antioxidant activities, *A. hydrophila* infection

In the published article, there was an error in affiliation(s) of the coauthor **Manal E. Shafi**.

The correct authorship and affiliations are as follows:

Seham El-Kassas^1*^, Nesreen Aljahdali^2^, Safaa E. Abdo^3^, Fatima S. Alaryani^4^, Eman M. Moustafa^5^, Radi Mohamed^6^, Wesam Abosheashaa^7^, Esraa Abdulraouf^7^, Mohamed Atef Helal^7^, Manal E. Shafi^8*^, Mohamed T. El-Saadony^9*^, Karima El-Naggar^10^ and Carlos Adam Conte-Junior^11^

^1^Animal, Poultry and Fish Breeding and Production, Department of Animal Wealth Development, Faculty of Veterinary Medicine, Kafrelsheikh University, Kafrelsheikh, Egypt

^2^Department of Biological Science, College of Science, King Abdulaziz University, Jeddah, Saudi Arabia

^3^Genetics and Genetic Engineering, Department of Animal Wealth Development, Faculty of Veterinary Medicine, Kafrelsheikh University, Kafrelsheikh, Egypt

^4^Biology Department, Faculty of Sciences, University of Jeddah, Jeddah, Saudi Arabia

^5^Department of Fish Diseases and Management, Faculty of Veterinary Medicine, Kafrelsheikh University, Kafrelsheikh, Egypt

^6^Department of Aquaculture, Faculty of Aquatic and Fisheries Sciences, Kafrelsheikh University, Kafrelsheikh, Egypt

^7^Department of Animal Wealth Development, Faculty of Veterinary Medicine, Kafrelsheikh University, Kafrelsheikh, Egypt

^8^Department of Biological Science, Zoology, Faculty of Science, King Abdulaziz University, Jeddah, Saudi Arabia

^9^Department of Agricultural Microbiology, Faculty of Agriculture, Zagazig University, Zagazig, Egypt

^10^Department of Nutrition and Veterinary Clinical Nutrition, Faculty of Veterinary Medicine, Alexandria University, Alexandria, Egypt

^11^Center for Food Analysis (NAL), Technological Development Support Laboratory (LADETEC), Federal University of Rio de Janeiro (UFRJ), Cidade Universitária, Rio de Janeiro, Brazil.

The authors apologize for this error and state that this does not change the scientific conclusions of the article in any way. The original article has been updated.

